# Supplementary material for: Learning about climate change uncertainty enables flexible water infrastructure planning
Source: Nat Commun. 2019 Apr 16;10:1782. doi: 10.1038/s41467-019-09677-x (PMC6468001; doi:10.1038/s41467-019-09677-x)
Supplement: Supplementary file 1 — Supplementary Information [file 41467_2019_9677_MOESM1_ESM.pdf]

Supplementary Information

Learning about climate change uncertainty enables flexible water infrastructure planning

Fletcher et al.

Supplementary Table 1

GCM ensembles used in Bayesian climate analysis

Modeling Center	Institute ID	Model Name (ens. member)
Commonwealth Scientific and Industrial Research Organization and Bureau of Meteorology, Australia	CSIRO/BOM	ACCESS 1.0 (1)<br>ACCESS 1.3 (1)
Beijing Climate Center, China Meteorological Administration	BCC	BCC-CSM1.1 (1)
EC-Earth Consortium	EC-EARTH	EC-EARTH (2,8,9,12)
The First Institute of Oceanography, SOA, China	FIO	FIO-ESM (2,3)
NOAA Geophysical Fluid Dynamics Laboratory	NOAA GFDL	GFDL-CM3 (1)<br>GFDL-ESM2G (1)<br>GFDL-ESM2M (1)
National Institute of Meteorological Research/Korea Meteorological Administration	NIMR/KMA	HadGEM2-AO (1)
Met Office Hadley Centre	MOHC	HadGEM2-CC (1)
Japan Agency for Marine-Earth Science and Technology, Atmosphere and Ocean Research Institute (The University of Tokyo), and National Institute for Environmental Studies	MIROC	MIROC-ESM-CHEM (1)<br>MIROC-ESM (1)
Atmosphere and Ocean Research Institute (The University of Tokyo), National Institute for Environmental Studies, and Japan Agency for Marine-Earth Science and Technology	MIROC	MIROC5 (1,2,3)
Norwegian Climate Centre	NCC	NorESM1-M (1)<br>NorESM1-ME (1)
