# Supplementary material for: Learning about climate change uncertainty enables flexible water infrastructure planning
Source: Nat Commun. 2019 Apr 16;10:1782. doi: 10.1038/s41467-019-09677-x (PMC6468001; doi:10.1038/s41467-019-09677-x)
Supplement: Supplementary file 3 — Description of Additional Supplementary Files [file 41467_2019_9677_MOESM3_ESM.pdf]

**Description of Additional Supplementary Information**

File Name: Supplementary Data 1

Description: Learning about climate change uncertainty enables flexible water infrastructure planning.
